# Successful identification of the species of the semipetrified amber medicinal resin benzoin using molecular diagnostic technology

**DOI:** 10.1038/s41598-023-30034-y

**Published:** 2023-02-20

**Authors:** Jian Feng, Qingqin He, Anzhen Xie, Yangyang Liu

**Affiliations:** 1 Hainan Provincial Key Laboratory of Resources Conservation and Development of Southern Medicine, Hainan Branch of the Institute of Medicinal Plant Development, Chinese Academy of Medical Sciences and Peking Union Medical College, No. 4, Yaogu 4th Road, Xiuying District, Haikou, 570311 Hainan Province China; 2 Key Laboratory of Bioactive Substances and Resources Utilization of Chinese Herbal Medicine, Ministry of Education and National Institute of Medicinal Plant Development, Chinese Academy of Medical Sciences and Peking Union Medical College, Beijing, 100193 China

**Keywords:** Plant molecular biology, DNA sequencing

## Abstract

Benzoin is an incomplete lithified resin secreted from the trunk of the *Styrax* Linn. that is known as "semipetrified amber" and has been widely used in medicine due to its blood circulation-promoting and pain-relieving properties. However, the lack of an effective species identification method due to the numerous sources of benzoin resin and the difficulty of DNA extraction has led to the uncertainty of species of benzoin in the trade process. Here, we report the successful extraction of DNA from benzoin resin containing bark-like residues and the evaluation of commercially available benzoin species using molecular diagnostic techniques. By performing a BLAST alignment of ITS2 primary sequences and homology prediction analysis of ITS2 secondary structures, we found that commercially available benzoin species were derived from *Styrax tonkinensis* (Pierre) Craib ex Hart. and *Styrax japonicus* Sieb. et Zucc. of the genus *Styrax* Linn. In addition, some of the benzoin samples were mixed with plant tissues from other genera, accounting for 29.6%. Therefore, this study provides a new method to solve the problem of species identification of semipetrified amber benzoin using information from bark residues.

## Introduction

Benzoin is the resin secreted from the trunk of the benzoin plant and is used as a medicine^1,2^. The main chemical components of benzoin are balsamic acids, lignans, terpenes, steroids, and other compounds^3–6^. Pharmacological studies have shown that benzoin possesses anti-inflammatory, antipyretic, antitumor, analgesic and estrogen-promoting properties, protects against cerebral hypoxia, and promotes blood–brain barrier permeability, among other properties^7–10^. Approximately 130 species of the genus *Styrax* Linn., both trees and shrubs, are distributed worldwide, mainly in Asia (China), Southeast Asia (Vietnam, Thailand, Sumatra, Indonesia) and North America^11,12^.

During the Tang Dynasty in ancient China, benzoin was recorded as a spice in the *Book of Jìn* and as a medicine in the *Tang Materia Medica* (*Xīnxiū Běn Cǎo*)^13,14^. It has a history of more than 700 years of medicinal use and was used in approximately 93 prescriptions, such as in *zhìbǎo boluse*s and *storax pill* in the *Tàimín Huìmín and Pharmaceutical Bureau Formula* and in *dàhuóluò boluses* in *Sages' Salvation Records*^15,16^. Benzoin has well-established uses in traditional forms of medicine. Several national pharmacopoeias, including those from China, the United States, Europe, and India, describe the specifications and tests for benzoin. The Chinese medicinal records identify benzoin as derived from the species *Styrax tonkinensis* (Pierre) Craib ex Hart., whereas *S. tonkinensis* and *Styrax benzoin* Dryander are recorded in the European Pharmacopoeia standard. The United States Pharmacopoeia and the Indian Pharmacopoeia record benzoin as derived from *S. benzoin*, *S. paralleloneurus* Perkins (trade name Sumatran benzoin), *S. tonkinensis*, or other species of the genus *Styrax* Linn. (trade name Siam benzoin)^17–21^. Benzoin is used in the form of a tincture^22^. The British Pharmacopoeia specifies the use of Sumatra benzoin in Benzoin Inhalation and Compound Benzoin Tincture^23^. The United States Pharmacopoeia also describes a compound benzoin tincture, although it does not specify which type of benzoin is to be used. The Swiss Pharmacopoeia and the Indian Pharmacopoeia describe a simple benzoin tincture using Siam benzoin. Benzoin is also used in other medicinal preparations (both official and proprietary), such as over-the-counter cough suppressants, cold and flu preventatives, lotions, mouthwashes, and antibacterial powders^24^. It is likewise used as an additive in aromatherapy^25^. In addition, benzoin is also widely used in food, flavoring, and daily chemical products. The main role of benzoin in food is as a flavoring agent, and it is used to create the flavor of chocolate. In Denmark and Switzerland, benzoin is used as a flavoring agent for baked goods, which can fix other flavors and add spiciness^26,27^.

Benzoin is approved for use in food in the United States. As with all food additives, benzoin is subject to periodic review by the Joint FAO/WHO Expert Committee on Food Additives to assess its safety^28^. In addition, the largest use of benzoin, in terms of comparative quantities, is for incense substitutes in religious ceremonies and for the manufacture of spices. These fragrances are then blended and used in a wide range of end products, such as personal hygiene and care products and household products^5,29^. Siamese benzoin produces a lighter-colored extract than Sumatran benzoin for use in the fragrance, flavor, and pharmaceutical industries to prepare resins, tinctures, or other types of extracts^30^. Therefore, we infer that species differences in benzoin may influence its traditional use.

The resin of one or various plants in the genus *Styrax* Linn. has been used as "benzoin", such as in the United States Pharmacopoeia collections. In China, this medicinal material was mainly imported from abroad through the Silk Road, a practice that continues to this day. Currently, the true identity of the benzoin species available in the Chinese domestic herbal market is uncertain, and the classification of medicinal plants and spices in general is unclear. In addition, in the North Sumatra region of Indonesia, the benzoin resin production process involves first removing shrubs and parasitic plants from around the benzoin tree, tapping the bark, and then cutting the opening with a knife. After an interval of 3–4 months, the resin is cut, usually in summer and autumn, dried for 1–2 weeks, and finally cleaned (removal of excess bark). The collected benzoin is pooled together, sorted according to quality, and sold^31^. The whole process is not strictly standardized and confusion between different species of benzoin resin is evident^32^. Therefore, a method for the identification of benzoin species must be established.

In recent years, research on benzoin quality has mainly been based on the development of a chemical-based volatile component analysis and aromatic lipid content determination methods, such as gas chromatography-mass spectrometry, solid phase microextraction, headspace sampling, and high-performance liquid chromatography frit fast atom bombardment mass spectrometry^33,34^. In addition, the existing quality standards for medicinal materials record benzoin quality control indicators, including identification, total ash content, loss-on-drying, alcohol-insoluble matter and benzoic acid contents. These conventional indicators are mainly used for benzoin quality conformity testing, but they do not identify the species of benzoin.

DNA barcoding has received increasing attention as a rapid and accurate method of species identification that compares a standard DNA fragment from the species of interest with a library of DNA barcodes of known taxonomy. It is characterized by the fact that only one or a few suitable gene segments are selected to accurately identify most species of the entire genus and family, and it has high repeatability and stability^35–37^. Chen et al.^38^ established a DNA barcode identification system for traditional Chinese medicinal materials. The ITS2 sequence is short in length and easy to amplify. It has been widely used in systematic research as a part of ITS and has been confirmed to have the highest resolution in DNA barcodes^38^. However, at present, molecular biology studies examining benzoin are mainly focused on the chromosome size, ploidy, chloroplast genome, phylogeny, and geographic evolution^39–42^.

The key to species identification using molecular diagnostic approaches is to extract DNA from the target. Previous reports have not addressed the species identification of benzoin resinous medicinal materials. Benzoin is a resinous medicinal material secreted by injured *Styrax* trees. Theoretically, these resins do not contain the tissues of the benzoin tree, and DNA is difficult to extract. However, when investigating the actual status of commercially available benzoin, we fortunately found that the resin contains a bark-like residue. This residue may be the bark of the *Styrax* tree. This residue is produced using a procedure similar to the process of amber formation, but the resin is not completely lithified, and thus it is called semipetrified amber^43–45^. However, these residues stick to the resin, and the DNA is not easily extracted. Therefore, the DNA extracted from these residues must be explored and then ITS2 primary and secondary structures should be used as molecular diagnostic techniques to identify the species. This study provides a new method to solve the problem of identifying species producing the semipetrified amber benzoin medicinal material by obtaining original information on these residues. This report is the first to show that the correlation of different information may help determine the origin of benzoin.

## Results

### DNA concentration, sequence analysis and species identification

**Figure 1:**
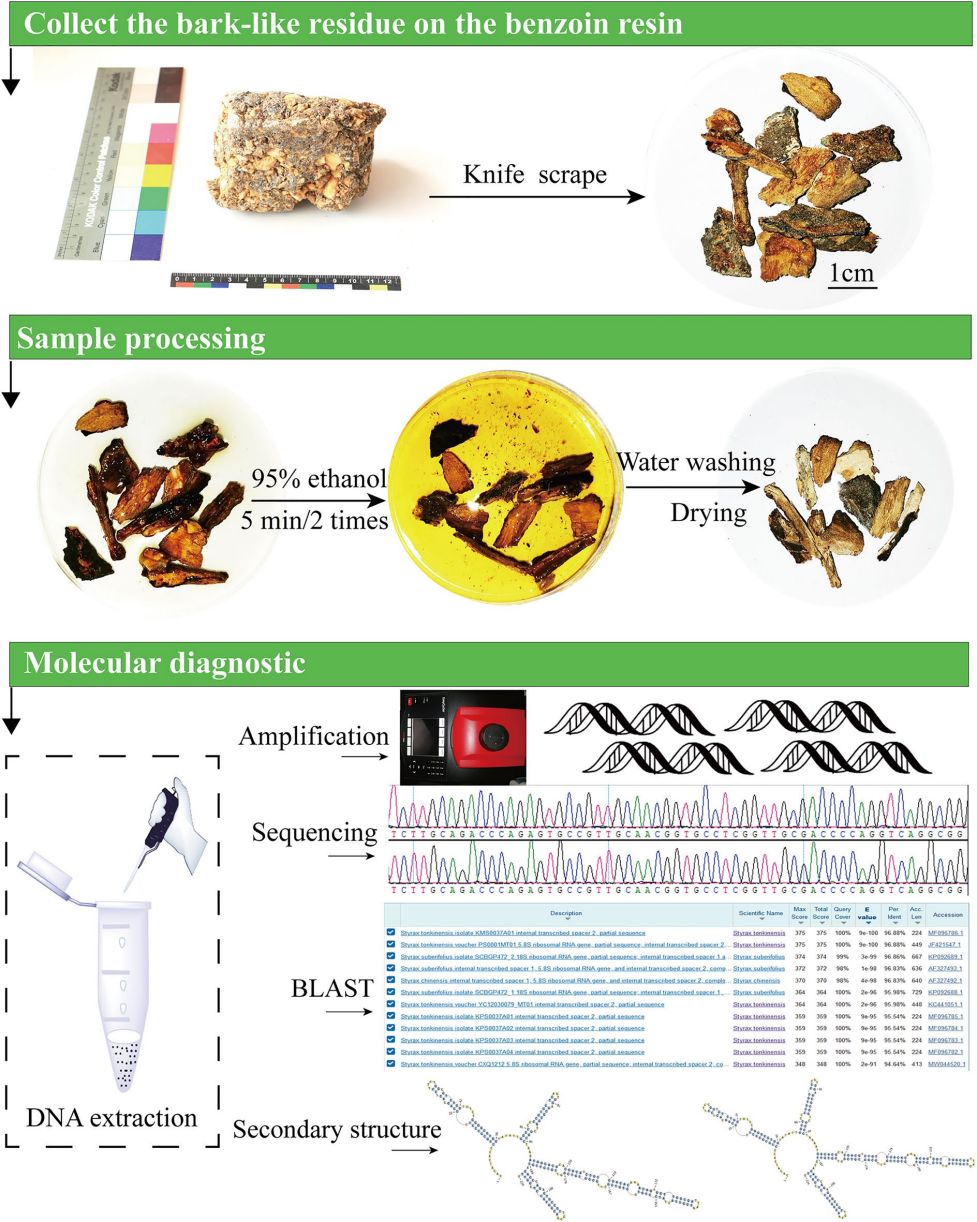
Process of pretreatment and molecular diagnostic identification of bark-like residues on benzoin.

We anticipated the direct extracting of DNA from crushed benzoin (resin) samples after sampling in this investigation, but the extracted DNA concentration was insufficient for subsequent tests (results not shown). Thus, we investigated scraping bark-like residues off the benzoin resin with a knife and treating each residue as a separate sample with molecular diagnostic identification, which has the advantage of preventing sequencing failure in subsequent trials due to sample mixing (Fig. 1).

In total, 27 batches of benzoin samples containing 40 individual bark-like residues were analyzed in this study. The DNA concentrations obtained from these residues ranged from 8.0 to 203.8 ng/μL, and the DNA purity ranged from 1.70 to 2.08. Although the AX151 sample had the lowest DNA concentration of 8.0 ng/μL, it had a purity of 1.89 and was able to be successfully amplified by PCR (Table 1). Therefore, treating the samples with 95% ethanol avoided the effect of resin on sample DNA extraction and amplification. In addition, because the samples originated from bark-like residues in benzoin resin, whose DNA was degraded, even though the DNA concentration and purity satisfied the requirements for subsequent experiments, we chose primers for the nuclear gene ITS2 as the universal barcode as primers for amplification, and all DNA samples successfully amplified and sequenced. The complete ITS2 sequence was obtained by removing the 5.8S and 26S regions from the sequences obtained using sequencing, which had a length of 217–230 bp.

**Table 1:** DNA concentration and purity of samples, and ITS2 length and GC content after sequencing.

Sample no	DNA concentration (ng/μL)	A260	A280	DNA purity (A260/A280)	ITS2 Length (bp)	GC content (%)
AX011	16.4	0.329	0.166	1.98	224	55.80
AX012	25.6	0.514	0.260	1.98	224	55.80
AX013	101.7	2.034	1.038	1.96	224	55.61
AX014	73.5	1.470	0.764	1.92	225	56.00
AX021	39.7	0.796	0.393	2.03	224	60.71
AX031	52.4	1.047	0.531	1.97	224	60.27
AX041	62.8	1.256	0.664	1.89	224	60.27
AX051	135.2	2.703	1.377	1.96	226	59.73
AX061	140.8	2.816	1.407	2.00	225	60.71
AX062	51.7	1.034	0.530	1.95	224	60.71
AX071	41.4	0.829	0.459	1.81	230	57.39
AX072	51.3	1.027	0.526	1.95	224	55.80
AX081	53.1	1.061	0.550	1.93	224	55.80
AX091	52.0	1.042	0.500	2.08	224	55.36
AX092	61.5	1.230	0.597	2.06	224	55.80
AX093	33.6	0.672	0.365	1.84	224	55.80
AX101	15.4	0.308	0.154	2.00	224	55.80
AX102	18.3	0.037	0.020	1.85	217	64.98
AX111	75.4	1.509	0.770	1.96	224	60.71
AX121	68.9	1.380	0.723	1.91	224	60.71
AX122	48.3	0.965	0.472	2.04	230	51.74
AX131	13.1	0.261	0.142	1.84	224	55.36
AX132	87.6	1.752	0.927	1.89	224	55.80
AX141	99.0	1.981	1.075	1.84	224	55.80
AX151	8.0	0.160	0.082	1.95	230	57.39
AX161	53.1	1.062	0.593	1.79	224	60.71
AX171	31.5	0.631	0.317	1.99	217	56.22
AX172	12.2	0.246	0.133	1.85	222	58.56
AX173	19.6	0.393	0.205	1.92	224	55.36
AX174	17.1	0.342	0.187	1.83	217	65.90
AX181	38.4	0.768	0.411	1.87	224	60.71
AX191	95.6	1.912	0.998	1.92	224	60.71
AX201	31.6	0.633	0.348	1.82	222	58.56
AX211	53.5	0.654	0.347	1.88	224	60.71
AX221	52.8	1.056	0.557	1.90	224	61.16
AX231	82.7	1.655	0.877	1.89	218	68.35
AX241	80.3	1.607	0.828	1.94	224	60.27
AX251	43.0	0.859	0.506	1.70	217	64.98
AX261	57.8	1.156	0.630	1.83	224	55.8
AX271	203.8	4.075	2.157	1.89	224	60.71

Forty individual samples were identified by a BLAST comparison with GenBank data from species of the genera *Styrax* Linn., *Dimocarpus* Lour., *Aquilaria* Lam., *Ageratum* L., *Glycine* Willd., *Acronychia* J. R. Forst. & G. Forst., *Trema* Lour., and *Musa* L., with a BLAST species identification rate > 92% (Table 2). The BLAST and secondary structure analyses showed that 30 samples were derived from the genus *Styrax* Linn., of which 16 were identified as *S. tonkinensis* and 14 were identified as *S. japonicus*. In addition, 10 samples were derived from other genera. The results from the BLAST search and secondary analysis results of sample AX174 were different, but both indicated that the sample belongs to the *Trema* Lour species. Samples AX071 and AX151 were identified as *Aquilaria sinensis* (Lour.) Spreng., AX102 and AX215 as *Dimocarpus longan* Lour., AX172 and AX201 as *Acronychia pedunculata* (L.) Miq., and AX171 as *Glycine soja* Siebold & Zucc. and *Glycine max* (Lour.) Merr. AX122 and AX231 were identified as *Ageratum conyzoides* L. and *Musa acuminata* Colla, respectively. These species are mainly found in tropical and subtropical regions, consistent with benzoin production areas. Benzoin is resinous, and it is inferred that the tissue of these plants may have been mixed with the harvested benzoin.

**Table 2:** Results of BLAST comparison and secondary structure analysis of ITS2 sequences of samples.

Sample no	BLAST results				Secondary structures results	
	*Styrax* Linn	Other species	Per. Ident (%)	E value	*Styrax* Linn	Other species
AX011	*S. japonicus*		99.55	9e−110	*S. japonicus*	
AX012	*S. tonkinensis*		99.55	1e−107	*S. tonkinensis*	
AX013	*S. japonicus*		99.55	9e−110	*S. japonicus*	
AX014	*S. japonicus*		99.55	9e−110	*S. japonicus*	
AX021	*S. tonkinensis*		96.88	9e−100	*S. tonkinensis*	
AX031	*S. tonkinensis*		94.20	1e−93	*S. tonkinensis*	
AX041	*S. tonkinensis*		96.88	3e−100	*S. tonkinensis*	
AX051	*S. tonkinensis*		92.40	6e−82	*S. tonkinensis*	
AX061	*S. tonkinensis*		96.88	9e−100	*S. tonkinensis*	
AX062	*S. tonkinensis*		96.88	9e−100	*S. tonkinensis*	
AX071		*Aquilaria sinensis* (Lour.) Spreng (*Aquilaria* Lam.)	99.13	2e−112		*A. sinensis*
AX072	*S. japonicus*		99.55	9e−110	*S. japonicus*	
AX081	*S. japonicus*		99.55	9e−110	*S. japonicus*	
AX091	*S. japonicus*		99.11	4e−108	*S. japonicus*	
AX092	*S. japonicus*		99.55	9e−110	*S. japonicus*	
AX093	*S. japonicus*		99.55	9e−110	*S. japonicus*	
AX101	*S. japonicus*		99.55	9e−110	*S. japonicus*	
AX102		*Dimocarpus longan* Lour (*Dimocarpus* Lour.)	100	1e-107		*D. longan*
AX111	*S. tonkinensis*		96.88	9e−100	*S. tonkinensis*	
AX121	*S. tonkinensis*		96.88	9e−100	*S. tonkinensis*	
AX122		*Ageratum conyzoides* L (*Ageratum* L.)	99.13	2e−112		*A. conyzoides*
AX131	*S. japonicus*		98.66	5e−107	*S. japonicus*	
AX132	*S. japonicus*		99.55	9e−110	*S. japonicus*	
AX141	*S. japonicus*		99.55	9e−110	*S. japonicus*	
AX151		*A. sinensis*	100	4e−113		*A. sinensis*
AX161	*S. tonkinensis*		96.88	9e−100	*S. tonkinensis*	
AX171		*Glycine soja* Siebold & Zucc (*Glycine* Willd.)	97.70	9e−100		*G. soja*
		*Glycine max* (L.) Merr (*Glycine* Willd.)	97.70	9e−100		*G. max*
AX172		*Acronychia pedunculata* (L.) Miq (*Acronychia* J. R. Forst. & G. Forst)	99.55	1e−108		*A. pedunculata*
AX173	*S. japonicus*		99.11	1e−108	*S. japonicus*	
AX174		*Trema cannabinum* var. dielsianum (Hand.Mazz.) C. J. Chen (*Trema* Lour.)	100	1e−107		*Trema micranthum* (L.) Blume
		*Trema nitidum* C. J. Chen (*Trema* Lour.)	100	1e−107		
		*Trema orientale* (L.) Blume (*Trema* Lour.)	100	1e−107		
		*Trema tomentosa* (Roxb.) H.Hara (*Trema* Lour.)	100	1e−107		
AX181	*S. tonkinensis*		96.88	9e−100	*S. tonkinensis*	
AX191	*S. tonkinensis*		96.88	9e−100	*S. tonkinensis*	
AX201		*Acronychia pedunculata* (L.) Miq (*Acronychia* J. R. Forst. & G. Forst.)	99.10	1e−107		*A. pedunculata*
AX211	*S. tonkinensis*		96.88	9e−100	*S. tonkinensis*	
AX221	*S. tonkinensis*		96.43	3e−99	*S. tonkinensis*	
AX231		*Musa acuminata* Colla *(Musa* L.)	96.33	1e−97		*M. acuminata*
AX241	*S. tonkinensis*		97.32	2e−101	*S. tonkinensis*	
AX251		*D. longan*	100	1e−107		*D. longan*
AX261	*S. japonicus*		99.55	9e−110	*S. japonicus*	
AX271	*S. tonkinensis*		96.88	9e−100	*S. tonkinensis*	

We assigned the 14 samples identified by BLAST as *S. japonicus* to group 1 and the 16 samples identified as *S. tonkinensis* to group 2. Twenty base variable loci were identified by ITS2 sequence alignment with *S. japonicus* and *S. tonkinensis* species in GenBank (Table 3). Base sites 13, 24, 46, 75, 78, 102, 104, 126, and 219 can be used as specific identification sites for these two species. However, different base sites were identified in the collected samples from these two species, such as G at base site 7 and C at base site 189 in group 2, while *S. japonicus* and *S. tonkinensis* species downloaded from GenBank had A and T for these base sites, respectively. In addition, two base sites in group 2 were identical to those of *S. japonicus* (base sites 32 and 209), and two base sites were variable (base sites 60 and 185).

**Table 3:** BLAST comparison of base difference sites of ITS2 sequences from *Styrax* Linn. samples with *S. japonicus* and *S. tonkinensis*.

Species	Base site											
	7	13	24	32	33	46	60	75	77	78	84	102
*S. japonicus*^1)^	A	**T**	**A**	T	T	**T**	A	**T**	T	**A**	T	**A**
Group 1	A	**T**	**A**	T	T	**T**	A	**T**	T	**A**	C	**A**
*S. tonkinensis*^2)^	A	**C**	**C**	C	C/T	**C**	A	**C**	T/A/C	**G**	C	**G/A**
Group 2	G	**C**	**C**	T	T	**C**	G/A	**C**	C	**G**	C	**G**
Species	104	105	106	109	126	185	113	189	200	209	219	
*S. japonicus*^1)^	**G**	C	A	T	**G**	T	A	T	T	A	**T**	
Group 1	**G**	C	A	T	**G**	T	A	T	T	A	**T**	
*S. tonkinensis*^2)^	**A**	T/C	G/A	T/C	**A**	G	A/G	T/C	T/C	G/A	**C**	
Group 2	**A**	C	A	C	**A**	G/C	A	C	C	A	**C**	

^1)^*S. japonicus* has the following accession numbers in Genbank: AB114900, LC534305, LC600960, and MF349070; ^2)^*S. tonkinensis* has the following accession numbers in Genbank: JF421547, MF096782, MF096783, MF096784, MF096785, and MF096786. Group 1 is the 14 samples identified as *S. japonicus* and group 2 is the 16 samples identified as *S. tonkinensis*. Bolded black indicates differential loci for *S. japonicus* and *S. tonkinensis.*

### Analysis of the ITS2 secondary structure

**Figure 2:**
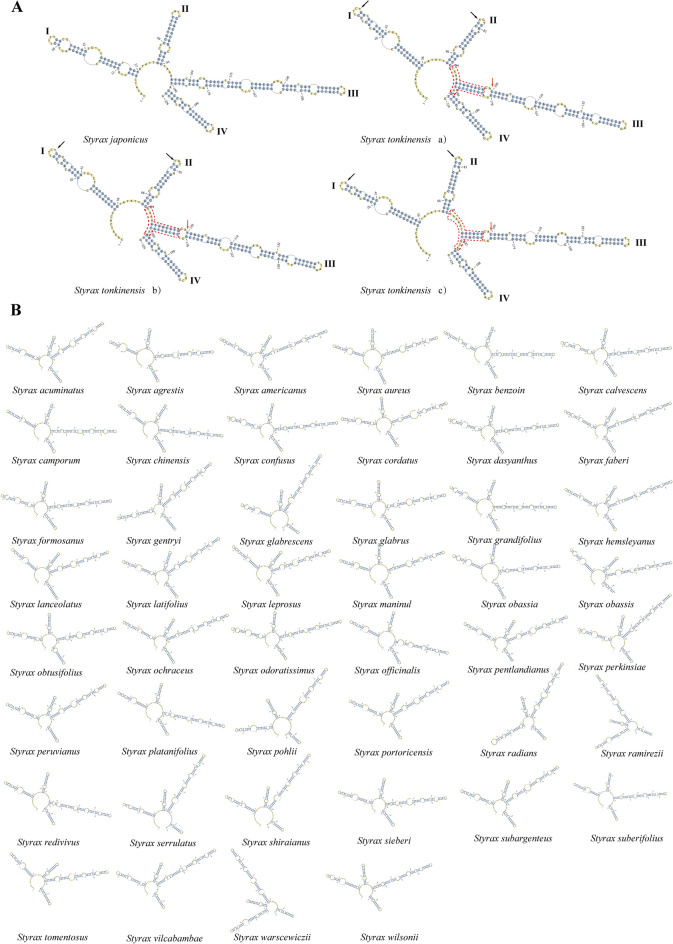
(**A**) Comparison of the secondary structure of ITS2 between benzoin samples from *S. tonkinensis* and *S. japonicus*. *S. tonkinensis* secondary structures a), b), and c) were all slightly different. Arrows indicate the sites of hCBCs (red) and different base sites (black), and the red dashed boxes represent the different base pairs in helix III of *S. tonkinensis*. (**B**) The secondary structures of ITS2 in 46 taxa analyzed in this study, except for *S. tonkinensis* and *S. japonicus*. The species name is marked below each structure.

The ITS2 database was used to predict the homology of the secondary structures of 48 major species of the genus *Styrax* Linn. The ITS2 secondary structures of all species were folded into the typical structure of a central loop with four helices (I, II, III, and IV), with helix III identified as the longest helix, followed by helices I, II, and IV. Helix II was the most conserved, with the central main loop relatively conserved and rich in purine bases^46–48^. The ITS2 secondary structure predictions for 40 individual samples are shown in Table 4. By homology prediction, 30 samples were derived from species of the genus *Styrax* Linn., 16 of which were identified as *S. tonkinensis* and 14 as *S. japonicus*. In addition, 10 samples were derived from species of other genera. The ITS2 secondary structures of the two species identified as *S. tonkinensis* and *S. japonicus* in the benzoin samples are shown in Fig. 2A. The helix positions and angles of the secondary structures of these two species are completely different, with helices I and III identified as more variable due to multiple unpaired bases and positional differences. In contrast, the shapes of helices II and IV, although basically the same, also contain unpaired bases, leading to differences. *S. tonkinensis* contains three slightly different secondary structures, as shown in Fig. 2A a), b), and c). The red dashed boxes represent the base pairs between the base of helix III and the first unpaired loop on the helix arm of *S. tonkinensis* a) and *S. tonkinensis* b), and these differences are due to differences in the position of helix III. *S. tonkinensis* contains three ITS2 secondary structures with different base sites, such as bases A, C, and T at position 32 in helix I (black arrows). In addition, hemi compensatory base changes (hCBCs) were identified in helix III (109/179: C-G → T-G, red arrows). We compared the DNA sequences of the two sets of basal samples obtained using BLAST identification. The ITS2 secondary structure sequence in group 1 was identical to that of *S. japonicus*, with the presence of hCBCs (73/84: G-C → G-T) detected only on helix II (Table 4). The results for the predicted secondary structure of ITS2 in group 2 homologs were the same as those of *S. tonkinensis*, but the differences in the position of helix III were clear, and variations in base sites were also observed. For example, sample AX051 differed from the other 15 samples by the secondary structure at base 60 in the main loop (A) and the presence of hCBCs in helix III (103–185: C-C → C-G), but the remaining sites were identical. Compared with *S. tonkinensis* a) and *S. tonkinensis* b), the bases at sites 7 and 60 of the main loop in group 2 are G, the bases at site 32 of the top asymmetric loop in helix I are T, the bases at site 77 of the top asymmetric loop of helix II are C, while the base 100/188 of helix III lacks the A-T base pair and has hCBCs (103–185: C-C → C-G), and base 209 of the top asymmetric loop of helix IV is A. Compared to *S. tonkinensis* c), the sample in group 2 have G bases at sites 7 and 60 of the main loop, T-T bases at sites 32–33 of the top asymmetric loop of helix I, different base pairs at 100–106/182–188 of helix III, and the presence of hCBCs on helix IV (200–215: T-G → C-G), as shown in Table 4. Therefore, the secondary structures of ITS2 from *S. tonkinensis* and *S. japonicus* differ significantly, and the species may be further precisely identified based on this information. In addition, the ITS2 secondary structures from the remaining 46 species of the genus *Styrax* Linn. are shown in Fig. 2B. The sizes, positions, and angles of the four helices and the bases in the helix vary among the 38 species, except for the four species pairs of *S. calvescens* and *S. formosanus*, *S. formosanus* and *S. dasyanthus*, *S. formosanus* and *S. confusus*, and *S. confusus* and *S. hemsleyanus*. The primary sequences in these four groups of species are identical to each other. We tried to analyze the differences in their secondary structures by measuring homologous folding, but unfortunately, the ITS2 secondary structures of the four groups of species were also identical.

**Table 4:** Comparison of base differences on the helices of *S. tonkinensis* and *S. japonicus* with the samples.

Species	Helix I	Helix II	Helix III	Helix IV	Main loop
*S. japonicus*	–	hCBCs (73/84: G-C → G-T)	–	–	–
Group 1					
*S. tonkinensis* a)	32: T → C	77: C → A; C → T	Position difference; 100/188:- → A-T hCBCs (103–185: C–C → C-G)	209: A → G	7: G → A; 60: G → A
*S. tonkinensis* b)					
Group 2					
*S. tonkinensis* c)	32/33:T-T → C–C	–	Position difference; 100–106/182–188: Base pairs difference	hCBCs (200–215: T-G → C-G)	7: G → A; 60: G → A
Group 2					

Group 1 was the 14 samples identified as *S. japonicus*, and group 2 was the 16 samples identified as S*. tonkinensis* by BLAST. –: represents no differences in base sequences.

The species of the 27 batches of benzoin samples collected were inferred from a comprehensive BLAST analysis and by analyzing the ITS2 secondary structures of 40 individual samples, as shown in Table 5. The AX01 sample contained two species of the genus *Styrax* Linn., *S. japonicus* and *S. tonkinensis*. No species of the genus *Styrax* Linn were detected in the four batches of samples (AX15, AX20, AX23, and AX25), and only other plant species were detected, but this result does not mean that these samples were not benzoin. The contamination of commercially available benzoin samples was prominent, accounting for 29.6% of the samples, which is related to the harvesting and transportation processes at the site of origin.

**Table 5:** Comprehensive determination of species in 27 batches of benzoin samples.

Batch no	Included species	Judgment results
AX01	*S. japonicus*; *S. tonkinensis*	Y
AX02	*S. tonkinensis*	Y
AX03	*S. tonkinensis*	Y
AX04	*S. tonkinensis*	Y
AX05	*S. tonkinensis*	Y
AX06	*S. tonkinensis*	Y
AX07	*S. japonicus*; *A. sinensis*	Y/N
AX08	*S. japonicus*	Y
AX09	*S. japonicus*	Y
AX10	*S. japonicus*; *D. longan*	Y/N
AX11	*S. tonkinensis*	Y
AX12	*S. tonkinensis*; *A. conyzoides*	Y/N
AX13	*S. japonicus*	Y
AX14	*S. japonicus*	Y
AX15	*A. sinensis*	–/N
AX16	*S. tonkinensis*	Y
AX17	*S. japonicus*; *G. soja*; *G. max*; *A. pedunculata*; *Trema* Lour	Y/N
AX18	*S. tonkinensis*	Y
AX19	*S. tonkinensis*	Y
AX20	*A. pedunculata*	–/N
AX21	*S. tonkinensis*	Y
AX22	*S. tonkinensis*	Y
AX23	*M. acuminata*	–/N
AX24	*S. tonkinensis*	Y
AX25	*D. longan*	–/N
AX26	*S. japonicus*	Y
ZX27	*S. tonkinensis*	Y

Y represents *Styrax* Linn. species, N represents other genus species, and – represents *Styrax* Linn. species not detected.

## Discussion

The core of Chinese medicine identification is to conduct variety authenticity and quality evaluation, which are related to drug safety and the quality assurance of the Chinese medicine industry. Traditional identification, as represented by the application of classical morphological classification to study the origin of Chinese herbs, is based on the description of individual traits and macroscopic observation, and the conclusions it yields are often imperfect^49^. This limitation is attributed to the need for specialized taxonomic knowledge, which is mostly based on an empirical judgment. In addition, microscopic identification, physicochemical identification, and other mainstream methods for the identification of Chinese medicinal materials have some limitations in the identification of genera with various species. For example, microscopic identification requires specialized expertise, and physical and chemical identification is time-consuming^50^. Molecular diagnostic technology, as represented by DNA barcoding, can accurately identify and characterize species and is a simple and precise method for the identification of Chinese medicinal materials with clear judgment criteria; it is thus suitable for the accurate identification of Chinese medicinal materials and their various species without requiring professional knowledge of taxonomy or ambiguous morphological characters^36^.

Most Chinese herbs and tablets undergo further processing and preparation steps, where DNA degradation is more severe, particularly for resinous herbs such as benzoin, which is formed by the solidification of secretions after the injury of trees of the genus *Styrax* Linn., and these resins mainly contain balsamic acids, lignans, and terpenoids^1,5,51–53^. Despite the original processing of the benzoin resin, bark or plant tissue fragments remain on the resin. However, these resins are insoluble in water, which increases the difficulty of DNA extraction. Therefore, the most critical step for molecular diagnostic techniques is to extract higher quality DNA from the samples. In preliminary experiments, benzoin was directly ground to extract DNA (the purity and concentration of most of the DNA samples were low and did not meet the experimental requirements), but the amplification results were unsatisfactory (most of the amplifications failed, and some sequences were heterozygous). This failure might be due to low DNA yield because of lower retention or resin inhibition. Therefore, we changed our approach and collected the bark-like residues directly from the benzoin resin and used each piece of residue as a separate sample. These bark-like residues were treated with 95% ethanol, and fortunately, DNA was successfully extracted and purified from them using a plant kit. The purpose of the 95% ethanol treatment is to remove as many of the substances that interfere with DNA release as possible (including other impurities). Previous scholars have mainly explored methods for extracting DNA extraction methods from dried wood (Lobeliaceae), archaeological wood remains, ancient wood (oak), endangered wood (prismatic wood), and other samples. Although the DNA of wood may be severely degraded, DNA can be extracted through method optimization^54–57^. To date, resinous medicinal materials have been studied in *Draconis sanguis* (resin exuded from fruits) and agarwood (wood-containing resin) by improving DNA extraction methods^58,59^. The present study is also the first to report the successful extraction of DNA from resin samples exuded from excised trees that meets the requirements for subsequent experiments. Therefore, this study shows that resinous materials used for DNA extraction must be pretreated.

In recent years, DNA barcodes have been widely used in species identification studies of medicinal materials, and the candidate barcodes are mostly the chloroplast markers *mat*K, *rbc*L, *psb*A-*trn*H, *trn*L, and *trn*K, the nuclear genes ITS and ITS2, and their corresponding barcode combinations^38,60–62^. However, for some samples with severe DNA degradation and fragmented DNA, the use barcodes with longer amplification products as primers is unsuccessful. The selection of barcodes with appropriate PCR amplification product lengths is crucial for molecular diagnostic studies, especially for DNA degradation or fragmentation in medical materials such as resinous or stemmed wood. ITS2, a nuclear gene sequence, is generally 200–300 bp in length and is suitable for identification at the genus and species levels. Moreover, the secondary structure is more conserved, which is a neck-loop structure formed by the single-strand DNA folding back on itself, with paired bases forming the stem and unpaired bases forming the loop, and a mature ITS2 database and structure prediction software are available^63,64^. In species identification studies, the prediction of secondary structure may be a useful complement to the primary structure phylogenetic tree by avoiding or excluding the misleading effect of paralogous homologs or pseudogenes on the primary structure^65^. Although the assessment of the ITS2 secondary structure was an early technique used for molecular diagnostics, it is still valid for species identification as a complement to DNA barcoding.

In the present study, 40 individual bark-like residues from 27 batches of benzoin were analyzed by performing BLAST searches of their ITS2 primary structure and secondary structure, and 30 were found to be from the benzoin genus and identified as *S. tonkinensis* and *S. japonicus*. Ten samples were identified as species of other genera, perhaps possibly related to mixing during resin collection and transport at the original cultivation site. However, uncertainty also exists about the results of identifying species of other genera in benzoin samples because only ITS2 was used as the barcode sequence in this study, and the reliable information obtained was limited. This study only presents the possibility of this potential contamination. Therefore, some additional primers (e.g., sequences of the chloroplast genome) are needed at a later stage to obtain more genetic information and improve the reliability of the identification results. In addition, morphological, taxonomic, or anatomical methods must be combined with comparative studies of the tissues of these species growing in the field to more accurately confirm the species information contained in the bark-like residues.

In this study, using the GenBank database, we compared the ITS2 sequences of the two species identified as benzoin, and 9 base sites were useful as specific identification sites for these two species. Therefore, this study is expected to develop specific DNA probes for the accurate identification of species of the genus Styrax Linn., which provides a good basis for determining the authenticity of benzoin products. Overall, this study provides a new method to solve the problem of species identification of the semipetrified amber medicinal resin benzoin by obtaining original information on these residues.

## Materials and methods

### Sample collection

Twenty-seven batches of benzoin medicinal materials were collected from medicinal material markets and drugstores in China. These collections are permitted and legal. All benzoin samples were identified by Professor Yangyang Liu of the Hainan Branch of the Institute of Medicinal Plants, Chinese Academy of Medical Sciences. We observed the visual properties of the collected benzoin samples and found that the samples were irregular and often agglomerated into clumps. The surface was orange–yellow or yellowish white, and the material was fragrant, with a gritty feel when chewed. It was very brittle and could be broken or cracked by hand; and the resin contained bark residues inside. Two to four replicates were added during the experiment for batches with high bark residues. Therefore, 40 individual bark-like residues from the final experimental materials were obtained, and the details are shown in Table 6. All voucher specimens of bark-like residues are maintained in the herbarium of the Aromatic Southern Medicine Identification Center, Hainan Branch, Institute of Medicinal Plants, Chinese Academy of Medical Sciences. In addition, 56 ITS2 sequences of 48 species of *Styrax* Linn. were downloaded from GenBank for comparative analysis (Table 7).

**Table 6:** Information on samples of benzoin was collected from medicinal markets and drugstores.

Batch no	Sample no	Label	Species	Producing area	Collection location	Purchase time
AX01	AX011	Benzoin	Unknown	Myanmar	Medicinal materials market of Anguo, China	2019.08.23
	AX012					
	AX013					
	AX014					
AX02	AX021	Benzoin	Unknown	Myanma		
AX03	AX031	Benzoin	Unknown	Myanma		
AX04	AX041	Benzoin	Unknown	Thailand		
AX05	AX051	Benzoin	Unknown	Iran		
AX06	AX061	Benzoin	Unknown	Iran		
	AX062					
AX07	AX071	Benzoin	Unknown	Vietnam		
	AX072					
AX08	AX081	Benzoin	Unknown	Myanmar	Medicinal materials market of Bozhou, China	2019.08.20
AX09	AX091	Benzoin	Unknown	Sumatra		
	AX092					
	AX093					
AX10	AX101	Benzoin	Unknown	Thailand		
	AX102					
AX11	AX111	Benzoin	Unknown	Indonesia		
AX12	AX121	Benzoin	Unknown	Indonesia		
	AX122					
AX13	AX131	Benzoin	Unknown	Laos	Medicinal materials market of Yulin, China	2019.09.26
	AX132					
AX14	AX141	Benzoin	Unknown	Laos		
AX15	AX151	Benzoin	Unknown	Laos		
AX16	AX161	Benzoin	Unknown	Uncertainty		
AX17	AX171	Benzoin	Unknown	Nepal	Medicinal materials market of Hehuachi, China	2019.12.14
	AX172					
	AX173					
	AX174					
AX18	AX181	Benzoin	Unknown	Indonesia		
AX19	AX191	Benzoin	Unknown	Uncertainty		
AX20	AX201	Benzoin	Unknown	India		
AX21	AX211	Benzoin	Unknown	Uncertainty		
AX22	AX221	Benzoin	Unknown	India		
AX23	AX231	Benzoin	Unknown	Uncertainty		
AX24	AX241	Benzoin	Unknown	Indonesia		
AX25	AX251	Benzoin	Unknown	Uncertainty		
AX26	AX261	Benzoin	Unknown	Indonesia		
AX27	AX271	Benzoin	Unknown	Uncertainty	Drugstore of Haikou, China	2019.6.20

**Table 7:** ITS2 sequences of species of the genus *Styrax* Linn. in Genbank.

No	Species	Genus	Collection site	Accession ID
1	*Styrax acuminatus* Pohl	*Styrax* Linn	Genbank	KP793409
2	*Styrax agrestis* (Lour.) G. Don	*Styrax* Linn	Genbank	AF327483
3	*Styrax americanus* Lam	*Styrax* Linn	Genbank	AF327472
4	*Styrax aureus* (Mart.) Miers	*Styrax* Linn	Genbank	AY143577
5	*Styrax benzoin* Dryander	*Styrax* Linn	Genbank	AF327494
6	*Styrax calvescens* Perk	*Styrax* Linn	Genbank	AF327468
7	*Styrax camporum* Pohl	*Styrax* Linn	Genbank	AF327504
8	*Styrax chinensis* Hu et S. Y. Liang	*Styrax* Linn	Genbank	AF327492
9	*Styrax confusus* Hemsl	*Styrax* Linn	Genbank	AF327470
10	*Styrax cordatus* A.D C	*Styrax* Linn	Genbank	AF327506
11	*Styrax dasyanthus* Perk	*Styrax* Linn	Genbank	AF327467
12	*Styrax faberi* Perk	*Styrax* Linn	Genbank	AF327485
13	*Styrax formosanus* Matsum	*Styrax* Linn	Genbank	AF327466
14	*Styrax gentryi* P.W. Fritsch	*Styrax* Linn	Genbank	AF327502
15	*Styrax glabrescens* Benth	*Styrax* Linn	Genbank	AF327475
16	*Styrax glabrus* Sw	*Styrax* Linn	Genbank	AY143582
17	*Styrax grandifolius* Ait	*Styrax* Linn	Genbank	AF327476
18	*Styrax hemsleyanus* Diels	*Styrax* Linn	Genbank	AF327477
19	*Styrax japonicus* Sieb. et Zucc	*Styrax* Linn	Genbank	AB114900; LC534305; LC600960; MF349070
20	*Styrax lanceolatus*P.W. Fritsch	*Styrax* Linn	Genbank	AF327501
21	*Styrax latifolius* Pohl	*Styrax* Linn	Genbank	AY143585
22	*Styrax leprosus* Hook. & Arn	*Styrax* Linn	Genbank	KP793405
23	*Styrax maninul* Hook. & Arn	*Styrax* Linn	Genbank	AY143576
24	*Styrax obassia* Siebold & Zucc	*Styrax* Linn	Genbank	AF327479
25	*Styrax obassis* Siebold et Zucc	*Styrax* Linn	Genbank	MF349081
26	*Styrax obtusifolius Griseb*	*Styrax* Linn	Genbank	AY143589
27	*Styrax ochraceus* Urb	*Styrax* Linn	Genbank	AY143580
28	*Styrax odoratissimus* Champ. ex Bentham	*Styrax* Linn	Genbank	AF327460
29	*Styrax officinalis* L	*Styrax* Linn	Genbank	AF327489
30	*Styrax pentlandianus* J. Rémy	*Styrax* Linn	Genbank	AF327507
31	*Styrax perkinsiae* Rehd	*Styrax* Linn	Genbank	AF327459
32	*Styrax peruvianus* Zahlbr	*Styrax* Linn	Genbank	KP793406
33	*Styrax platanifolius* Engelm. ex Torr	*Styrax* Linn	Genbank	AF327486
34	*Styrax pohlii* A. D C	*Styrax* Linn	Genbank	AY143581
35	*Styrax portoricensis* Krug & Urban	*Styrax* Linn	Genbank	AF327505
36	*Styrax radians* P.W. Fritsch	*Styrax* Linn	Genbank	AF327497
37	*Styrax ramirezii* Greenm	*Styrax* Linn	Genbank	AF327499
38	*Styrax redivivus* (Torr.) L.C. Wheeler	*Styrax* Linn	Genbank	AF327490
39	*Styrax serrulatus* Roxb	*Styrax* Linn	Genbank	AF327481
40	*Styrax shiraianus* Makino	*Styrax* Linn	Genbank	AF327480
41	*Styrax sieberi* Makino	*Styrax* Linn	Genbank	AY143587
42	*Styrax subargenteus* Sleumer	*Styrax* Linn	Genbank	KP793407
43	*Styrax suberifolius* Hook. et Arn	*Styrax* Linn	Genbank	AF327493
44	*Styrax tomentosus* Bonpl	*Styrax* Linn	Genbank	AY143590
45	*Styrax tonkinensis* (Pierre) Craib ex Hartw	*Styrax* Linn	Genbank	MF096782; MF096783; MF096784; MF096785; JF421547; MF096786;
46	*Styrax vilcabambae* (D.R. Simpson) B. Walln	*Styrax* Linn	Genbank	AF327496
47	*Styrax warscewiczii* Perkins	*Styrax* Linn	Genbank	AF327500
48	*Styrax wilsonii* Rehd	*Styrax* Linn	Genbank	AF327462

### DNA extraction, amplification, and sequencing

A knife was used to scrape and collect the bark-like residue in the benzoin resin, which was placed in disposable petri dishes. An appropriate amount of 95% ethanol was added twice and incubated for 5 min each, and the residue was finally washed with sterile water and dried (Fig. 1). The sample was cut into pieces and placed in a 2 mL centrifuge tube to which two steel balls were added. Then, it was frozen in liquid nitrogen and ground in a high-throughput tissue grinder for 5 min. DNA was extracted with the modified HP Plant DNA kit (OMEGA Bio-tek), where 1000 μL of buffer CPL and 10 μL of β-mercaptoethanol were added, and the remaining steps were performed according to the manufacturer’s instructions. DNA samples were purified using the MicroElute® DNA Clean-Up Kit. The concentration of total DNA was measured, the DNA purity (the ratio of A260 to A280) was calculated, and the extracted DNA was stored in a − 20 °C freezer until use. Amplification was performed using primers for ITS2 (ITS2-F: ATGCGATACTTGGTGTGAAT; ITS2-R: GACGCTTCTCCAGACTACAAT)^39^. The PCR amplification program was as follows: predenaturation at 94 °C for 5 min; 40 cycles of denaturation at 94 °C for 30 s, annealing at 56 °C for 30 s, and extension at 72 °C for 45 s; extension at 72 °C for 10 min; and storage at − 20 °C. PCR products were sequenced bidirectionally at Guangzhou Aike Biotechnology Co., Ltd. (Guangzhou, China).

### Data analysis

Sequence splicing was performed using CodonCode Aligner V3.7.1 (CodonCode Co., USA) to remove primer regions and low-quality sequences. The sequences obtained by sequencing and those downloaded from GenBank were annotated and cropped. Based on hidden Markov models (HMMs), the 5.8S and 26S regions were removed to obtain the complete ITS2 sequence^63^. The complete ITS2 sequence obtained through sequencing was used for the BLAST alignment analysis in GenBank, and MEGA 6.0 was used for alignment. The variation sites were recorded, and the species information of the samples was analyzed. Moreover, the ITS2 secondary structures of all species were predicted using the ITS2 database homology model^64^. Additionally, hCBCs (such as C-G → C-A or T-T → T-C) were calculated.

## Supplementary Information

Supplementary Information.

## Data Availability

All the data generated during this study are included in this published article and its supplementary information files. ITS2 sequences generated in this study are available at GenBank (accession numbers: OP787212-OP787251). The 56 ITS2 sequences of *Styrax* Linn. were downloaded from GenBank for comparative analysis, accession no: KP793405-KP793407, KP793409, MF096782-MF096786, MF349070, MF349081, AY143580-AY143582, AY143576, AY143577, AY143585, AY143587, AY143589, AY143590, JF421547, AB114900, LC534305, LC600960, AF327459, AF327460, AF327462, AF327466-AF327468, AF327470, AF327472, AF327475-AF327477, AF327479-AF327481, AF327483, AF327485, AF327486, AF327489, AF327490, AF327492-AF327494, AF327496, AF327497, AF327499, AF327500-AF327502, AF327504-AF327507.

## References

[1] Hacini Z, Khedja F, Habib I, Kendour Z, Debba Z (2018). Evaluation of antibacterial and antioxidant activities of three types of benzoin resin. Eur. J. Chem..

[2] Hu P (2016). Study on the Quality of Benzoin Based on Coniferyl Benzoate and Pharmacokinetics.

[3] Chen QF, Chen XZ, Li GY, Wang C, Zhang GL (2012). Two new 2-phenylbenzofurans from the bark of *Styrax*
*perkinsiae*. Chin. J. Nat. Med..

[4] Min BS, Cao TQ, Hung TM, Kim JA (2015). Inhibitory effect on no production of compounds from *Styrax*
*obassia*. Planta Med..

[5] Burger P (2016). New insights in the chemical composition of benzoin balsams. Food Chem..

[6] El-Razek MHA (2018). Triterpenes from *Styrax*
*benzoin*. Der Pharma Chemica..

[7] Kim MR (2004). Matrix metalloproteinase-1 inhibitor from the stem bark of *Styrax*
*japonica* S. et Z. Chem. Pharm. Bull..

[8] Kwon JH (2008). Triterpenoids and a sterol from the stem-bark of *Styrax*
*japonica* and their protein tyrosine phosphatase 1B inhibitory activities. Phytother. Res..

[9] Lee SJ, Lee J, Song S, Lim KT (2016). Glycoprotein isolated from *Styrax*
*japonica* Siebold et al. Zuccarini inhibits oxidative and pro-inflammatory responses in HCT116 colonic epithelial cells and dextran sulfate sodium-treated ICR mice. Food Chem. Toxicol..

[10] Gu AT, Zhang Q, Wang F (2017). A preliminary study on the active ingredient of *Styrax*
*tonkinensis* to protect endothelial injury. Gd Chem..

[11] IBCAS (1996). STYRAX Linnaeus. Flora China..

[12] Liu Z (2019). Revision of the Morphological Taxonomy of the Genus Styrax L. (Styracaceae) in China.

[13] Su J (2004). Tang materia medica. Anhui Sci. Tech..

[14] Fang XL (2008). The Book of Jìn. Zhonghua Book Publi. Press.

[15] Ji SY (2018). Formulaology. China TCM. Press.

[16] Chen SW (2005). Tàimín Huìmín and Pharmaceutical Bureau Formula. Shanghai People's Publi. Press.

[17] National Medical Products Administration. *Notice on Promulgating Quality Standards for 43 Kinds of Imported Medicinal Materials**, **Including Ercha*. 13. https://www.nmpa.gov.cn/xxgk/fgwj/gzwj/gzwjyp/20040508010101798.html (2004).

[18] EDQM Council of Europe. *European Pharmacopoeia EP10.0* 1341–1342 (2008).

[19] Indian Pharmacopoeia Commission. *Indian Pharmacopoeia* 888–889 (2010).

[20] Chinese Pharmacopoeia Commission (2020). Pharmacopoeia of the People’s Republic of China. China Med. Sci. Press.

[21] Kharidia, S. The United States Pharmacopeial. NF38. https://online.uspnf.com/uspnf/ (2020).

[22] Bedigian D (2003). Monograph on Benzoin (Balsamic resin from Styrax species). Econ. Bot..

[23] Medicines & healthcare products regulatory agency. British Pharmacopoeia, Vol. IV. *TSO*. 46–48. www.Pharmacopoeia.co.uk (2013).

[24] Sabbah DA (2018). Benzoin schiff bases: Design, synthesis, and biological evaluation as potential antitumor agents. Med. Chem..

[25] Scardamaglia L, Nixon R, Fewings J (2003). Compound tincture of benzoin: A common contact allergen?. Australas. J. Dermatol..

[26] Castel C (2006). Volatile constituents of benzoin gums: Siam and Sumatra, part 2. Study of headspace sampling methods. Flavour Fragr. J..

[27] Abdulmumeen HA, Risikat AN, Sururah AR (2012). Food: Its preservatives, additives, and applications. Int. J. Chem. Biol. Sci..

[28] Joint FAO/ WHO Expert Committee on Food Additives. *Evaluation of Certain Food Additives and Contaminants**: **Seventy-Fourth Report of the Joint FAO/WHO Expert Committee on Food Additives. WHO Technical Report Series* 966 (2011).

[29] Langenheim JH (2003). Plant Resins: Chemistry, Evolution Ecology and Ethnobotany.

[30] Murthy HN (2022). Gums, Resins and Latexes of Plant Origin, Reference Series in Phytochemistry.

[31] Harada K, Wiyono XX, Munthe L (2022). Production and commercialization of benzoin resin: Exploring the value of benzoin resin for local livelihoods in North Sumatra, Indonesia. Trees For. People..

[32] Sun YY, Feng J, Wu MS, Liu YY (2022). Herbal Textual research on benzoinum in famous classical formulas. Chin. J. Exp. Tradit. Med. Formulae.

[33] Mao D (2022). Authenticity assessment of (E)-cinnamic acid, vanillin, and benzoic acid from Sumatra benzoin balsam by gas chromatography combustion/pyrolysis isotope ratio mass spectrometry. Int. J. Anal. Chem..

[34] Fernandez X (2006). Volatile constituents of benzoin gums: Siam and Sumatra, part 3. Fast characterization with an electronic nose. Flavour Fragr. J..

[35] Gregory TR (2005). DNA barcoding does not compete with taxonomy. Nature.

[36] Michel CI, Meyer RS, Taveras Y, Molina J (2016). The nuclear internal transcribed spacer (ITS2) as a practical plant DNA barcode for herbal medicines. J. Appl. Res. Med. Aromat. Plants.

[37] Veldman S (2020). DNA barcoding augments conventional methods for identification of medicinal plant species traded at Tanzanian markets. J. Ethnopharmacol..

[38] Chen SL (2010). Validation of the ITS2 region as a novel DNA barcode for identifying medicinal plant species. PLoS ONE.

[39] Fritsch PW (2003). Multiple geographic origins of Antillean *Styrax*. Syst. Bot..

[40] Tong LL (2020). The complete chloroplast genome of *Styrax **faberi* Perk. (Styracaceae). Mitochondrial DNA Part B Resour..

[41] Cai XL (2021). Plastome structure and phylogenetic relationships of Styracaceae (Ericales). BMC Ecol. Evol..

[42] Song Y (2022). Chloroplast genome evolution and species identification of *Styrax* (Styracaceae). BioMed. Res. Int..

[43] Lobdell MS, Shearer K (2022). Genome sizes, ploidy levels, and base compositions of *Styrax* species and cultivars. HortScience.

[44] Valderrama NDL, Schaeffer P, Leprince A, Schmitt S, Adam P (2022). Novel oxygenated fossil nor-diterpenoids from Cretaceous amber (South-Western France) as potential markers from Cupressaceae and/or Cheirolepidiaceae. Org. Geochem..

[45] Gomez B, Martínez-Delclos X, Bamford M, Philippe M (2002). Taphonomy and palaeoecology of plant remains from the oldest African Early Cretaceous amber locality. Lethaia.

[46] Zhang ZQ (2021). Spectral characteristics of amber—their application in provenance determination, and study on fluorescent components. China Univ. Geosci..

[47] Schultz J (2005). A common core of secondary structure of the internal transcribed spacer 2 (ITS2) throughout the Eukaryota. RNA.

[48] Wolf M (2005). Homology modeling revealed more than 20,000 rRNA internal transcribed spacer 2 (ITS2) secondary structures. RNA.

[49] Ankenbrand MJ (2015). ITS2 database V: Twice as much. Mol. Biol. Evol..

[50] Huang LQ, Yuan Y (2014). Molecular identification of traditional Chinese medicine (TCM). Shanghai Sci. Tech. Publ..

[51] Ma SJ (2017). Molecular Identification of Herbal Medicines: From Barcode to Genomes.

[52] Li QL (2005). Four new benzofurans from seeds of *Styrax*
*perkinsiae*. Planta Med..

[53] Park SY (2007). Benzofurans from the seeds of *Styrax*
*obassia*. Bull. Korean Chem. Soc..

[54] Son NT (2021). Genus *Styrax*: A resource of bioactive compounds. Stud. Nat. Prod. Chem..

[55] Schwörer C, Leunda M, Alvarez N, Gugerli F, Sperisen C (2022). The untapped potential of macrofossils in ancient plant DNA research. New Phytol..

[56] Asif MJ, Cannon CH (2005). DNA extraction from processed wood A case study for the identification of an endangered timber species (*Gonystylus*
*bancanus*). Plant Mol. Biol. Rep..

[57] Liepelt S (2006). Authenticated DNA from ancient wood remains. Ann. Bot..

[58] Tnah LH (2012). DNA extraction from dry wood of *Neobalanocarpus*
*heimii* (Dipterocarpaceae) for forensic DNA profiling and timber tracking. Wood Sci. Technol..

[59] Wang J (2015). DNA barcodes identification of one rare traditional Chinese medicine *Draconis*
*sanguis*. Chin. Pharm. J..

[60] Jiao LC, Yin YF, Cheng YM, Jiang XM (2014). DNA barcoding for identification of the endangered species *Aquilaria*
*sinensis*: Comparison of data from heated or aged wood samples. Holzforschung.

[61] Nishizawa T, Watano Y (2000). Primer pairs suitable for PCR-SSCP analysis of chloroplast DNA in angiosperms. J. Phytogeogr. Taxon.

[62] Chung S, Decker-Walters DS, Staub JE (2003). Genetic relationships within the Cucurbitaceae as assessed by consensus chloroplast simple sequence repeats (ccSSR) marker and sequence analyses. Can. J. Bot..

[63] Hollingsworth ML (2009). Selecting barcoding loci for plants: Evaluation of seven candidate loci with species-level sampling in three divergent groups of land plants. Mol. Ecol. Resour..

[64] Keller A (2009). 5.8S–28S rRNA interaction and HMM-based ITS2 annotation. Gene.

[65] Koetschan C (2012). ITS2 database IV: Interactive taxon sampling for internal transcribed spacer 2 based phylogenies. Mol. Phylogenet. Evol..

