# Supplementary material for: Successful identification of the species of the semipetrified amber medicinal resin benzoin using molecular diagnostic technology
Source: Sci Rep. 2023 Feb 20;13:2943. doi: 10.1038/s41598-023-30034-y (PMC9941088; doi:10.1038/s41598-023-30034-y)
Supplement: Supplementary file 1 — Supplementary Information. [file 41598_2023_30034_MOESM1_ESM.docx]

**Supplementary information**

**Raw sequence and secondary structure results of the 40 individual bark-like residues in this study.**

**Raw sequence**

**AX011**

CACATCACCCTGTGCCCTCCCAGAACTCCTTTTGGCGTCTGGTGTTTGGGGCGTGGTATATTGGCCCCCCGTGCTCTATGGAGCGCGGTCGGCCTAAAATGACGCACATGACATCTGTCGACACAGAACAGTGGTTGGCGATTAGTCGTTACTATCCTGTGGTGTCGCTTGCGGTGTCGGTTGTTTTGTAGACCCAGAGTGCCGTTGCAACGGTGCCTTGGTTG

**AX012**

CACATCACCCTGTGCCCTCCCAGAACTCCTTTTGGCGTCTGGTGTTTGGGGCGTGGTATATTGGCCCCCCGTGCTCTATGGAGCGCGGTCGGCCTAAAATGACGCACATGACATCTGTCGACACAGAACAGTGGTTGGCGATTAGTCGTTACTATCCTGTGGTGTCGCTTGCGGTGTCGGTTGTTTTGTAGACCCAGAGTGCCGTTGCAACGGTGCCTTGGTTG

**AX013**

CACATCACCCTGTGCCCTCCCAGAACTCCTTTTGGCGTCTGGTGTTTGGGGCGTGGTATATTGGCCCCCCGTGCTCTATGGAGCGCGGTCGGCCTAAAATGACGCACATGACATCTGTCGACACAGAACAGTGGTTGGCGATTAGTCGTTACTATCCTGTGGTGTCGCTTGCGGTGTCGGTTGTTTTGTAGACCCAGAGTGCCGTTGCAACGGTGCCTTGGTTG

**AX014**

CACATCACCCTGTGCCCTCCCAGAACTCCTTTTGGCGTCTGGTGTTTGGGGCGTGGTATATTGGCCCCCCGTGCTCTATGGAGCGCGGTCGGCCTAAAATGACGCACATGACATCTGTCGACACAGAACAGTGGTTGGCGATTAGTCGTTACTATCCTGTGGTGTCGCTTGCGGTGTCGGTTGTTTTGTAGACCCAGAGTGCCGTTGCAACGGTGCCTTGGTTG

**AX021**

CACATCGCCCTGCGCCCTCCCAGCACTCCTTTTGGCGTCTGGTGTCTGGGGCGTGGTATGTTGGCCCCCCGTGCCCCGTGGAGCGCGGTCGGCCTAAAATGGCACACACGACATCTGTCGACACAAAACAGTGGTTGGCGATTAGTCGTTACTATCCTGTGGTGTCGCTTGCGGTGTCGGTTGTCTTGCAGACCCAGAGTGCCGTTGCAACGGTGCCTCGGTTG

**AX031**

CACATCGCCCTGCGCCCTCCCAGCACTCCTTTTGGCGTCTGGTGTCTGGGGCGTGGTATGTTGGCCCCCCGTGCCCCGTGGAGCGCGGTCGGCCTAAAATGGCACACACGACATCTGTCGACACAAAACAGTGGTTGGCGATTAGTCGTTACTATCCTGTGGTGTCGCTTGCGGTGTCGGTTGTCTTGCAGACCCAGAGTGCCGTTGCAACGGTGCCTCGGTTG

**AX041**

CACATCGCCCTGCGCCCTCCCAGCACTCCTTTTGGCGTCTGGTGTCTGGGGCGTGGTATATTGGCCCCCCGTGCCCCGTGGAGCGCGGTCGGCCTAAAATGGCACACACGACATCTGTCGACACAAAACAGTGGTTGGCGATTAGTCGTTACTATCCTGTGGTGTCGCTTGCGGTGTCGGTTGTCTTGCAGACCCAGAGTGCCGTTGCAACGGTGCCTCGGTTG

**AX051**

CACATCGCCCTGCGCCCTCCCAGCACTCCTTTTGGCGTCTGGTGTCTGGGGCGTGGTATATTGGCCCCCCGTGCCCCGTGGAGCGCGGTCGGCCTAAAATGGCACACACGACATCTGTCGACACAAAACAGTGGTTGGCGATTAGTCGTTACTATCCTGTGGTGTCGCTTGCGGTGTCGGTTGTGTTGCAGACCCAGAGTGCCGTTGCAACGGTGCCTCGGTTG

**AX061**

CACATCGCCCTGCGCCCTCCCAGCACTCCTTTTGGCGTCTGGTGTCTGGGGCGTGGTATGTTGGCCCCCCGTGCCCCGTGGAGCGCGGTCGGCCTAAAATGGCACACACGACATCTGTCGACACAAAACAGTGGTTGGCGATTAGTCGTTACTATCCTGTGGTGTCGCTTGCGGTGTCGGTTGTCTTGCAGACCCAGAGTGCCGTTGCAACGGTGCCTCGGTTG

**AX062**

CACATCGCCCTGCGCCCTCCCAGCACTCCTTTTGGCGTCTGGTGTCTGGGGCGTGGTATGTTGGCCCCCCGTGCCCCGTGGAGCGCGGTCGGCCTAAAATGGCACACACGACATCTGTCGACACAAAACAGTGGTTGGCGATTAGTCGTTACTATCCTGTGGTGTCGCTtGCGGTGTCGGTTGTCTTGCAGACCCAGAGTGCCGTTGCAACGGTGCCTCGGTTG

**AX071**

CGCATTGTAGCCCCCCACCCTCGTCGTAATGTCTGTGAGGGCTGTGTGGGGCTGATACTGGCCTTCCCGTATGCACAGCAATGCGGTTGGCCCAAATGGAGGAACCCAGGGCGGTGTATGCCWTGATGAACGGTGGTGTGTGCTTAGCCTGCCGTCGTTAGAGCATCATGCGCATCATGCCCTTGAGGTATGTTTYGTGGACAACCCCGGTGCAATCATAGCGCGCATCG

**AX072**

CACATCACCCTGTGCCCTCCCAGAACTCCTTTTGGCGTCTGGTGTTTGGGGCGTGGTATATTGGCCCCCCGTGCTCTATGGAGCGCGGTCGGCCTAAAATGACGCACATGACATCTGTCGACACAGAACAGTGGTTGGCGATTAGTCGTTACTATCCTGTGGTGTCGCTTGCGGTGTCGGTTGTTTTGTAGACCCAGAGTGCCGTTGCAACGGTGCCTTGGTTG

**AX081**

CACATCACCCTGTGCCCTCCCAGAACTCCTTTTGGCGTCTGGTGTTTGGGGCGTGGTATATTGGCCCCCCGTGCTCTATGGAGCGCGGTCGGCCTAAAATGACGCACATGACATCTGTCGACACAGAACAGTGGTTGGCGATTAGTCGTTACTATCCTGTGGTGTCGCTTGCGGTGTCGGTTGTTTTGTAGACCCAGAGTGCCGTTGCAACGGTGCCTTGGTTG

**AX091**

CACATCACCCTGTGCCCTCCCAGAACTCCTTTTGGCGTCTGGTGTTTGGGGCGTGGTATATTGGCCCCCCGTGCTCTATGGAGCGCGGTCGGCCTAAAATGACGCACATGACATCTGTCGACACAGAACAGTGGTTGGCGATTAGTCGTTACTATCCTGTGGTGTCGCTTGCGGTGTCGGTTGTTTTGTAGACCCAGAGTGCCGTTGCAACGGTGCCTTGGTTG

**AX092**

CACATCACCCTGTGCCCTCCCAGAACTCCTTTTGGCGTCTGGTGTTTGGGGCGTGGTATATTGGCCCCCCGTGCTCTATGGAGCGCGGTCGGCCTAAAATGACGCACATGACATCTGTCGACACAGAACAGTGGTTGGCGATTAGTCGTTACTATCCTGTGGTGTCGCTTGCGGTGTCGGTTGTTTTGTAGACCCAGAGTGCCGTTGCAACGGTGCCTTGGTTG

**AX093**

CACATCACCCTGTGCCCTCCCAGAACTCCTTTTGGCGTCTGGTGTTTGGGGCGTGGTATATTGGCCCCCCGTGCTCTATGGAGCGCGGTCGGCCTAAAATGACGCACATGACATCTGTCGACACAGAACAGTGGTTGGCGATTAGTCGTTACTATCCTGTGGTGTCGCTTGCGGTGTCGGTTGTTTTGTAGACCCAGAGTGCCGTTGCAACGGTGCCTTGGTTG

**AX101**

CACATCACCCTGTGCCCTCCCAGAACTCCTTTTGGCGTCTGGTGTTTGGGGCGTGGTATATTGGCCCCCCGTGCTCTATGGAGCGCGGTCGGCCTAAAATGACGCACATGACATCTGTCGACACAGAACAGTGGTTGGCGATTAGTCGTTACTATCCTGTGGTGTCGCTTGCGGTGTCGGTTGTTTTGTAGACCCAGAGTGCCGTTGCAACGGTGCCTTGGTTG

**AX102**

CGCATCGTCGCCCCCTCAACCCCACGCCTCGAGCGAGGCGCGGGGACACGTTAGGGGCGGATATTGGCCTCCCGTGGGACGCTTAATCCCGCGGTTGGCCCAAATACGAGTCCTCGGCGTCGGACGCCGCGACGTTCGGTGGTGGAAAAAGTAAGAAACCTCGAGCTCGCGTCGCGCGTACGTCGTCGGTTTAAGGCTCCCGACCCTGAACGCATCG

**AX111**

CACATCGCCCTGCGCCCTCCCAGCACTCCTTTTGGCGTCTGGTGTCTGGGGCGTGGTATGTTGGCCCCCCGTGCCCCGTGGAGCGCGGTCGGCCTAAAATGGCACACACGACATCTGTCGACACAAAACAGTGGTTGGCGATTAGTCGTTACTATCCTGTGGTGTCGCTTGCGGTGTCGGTTGTCTTGCAGACCCAGAGTGCCGTTGCAACGGTGCCTCGGTTG

**AX121**

CACATCGCCCTGCGCCCTCCCAGCACTCCTTTTGGCGTCTGGTGTCTGGGGCGTGGTATGTTGGCCCCCCGTGCCCCGTGGAGCGCGGTCGGCCTAAAATGGCACACACGACATCTGTCGACACAAAACAGTGGTTGGCGATTAGTCGTTACTATCCTGTGGTGTCGCTTGCGGTGTCGGTTGTCTTGCAGACCCAGAGTGCCGTTGCAACGGTGCCTCGGTTG

**AX122**

CGCATCACGTCGCCCGCGTCACACATCCTTTCTTGGATTGTGTTGTACGTGGGCGGATACTGGTCTTCCGTGCCCATGGTGTGGTTGGCCCAAACAGGAGTCCGCTAAAGAAAGAGGCACGACTGGTGGTGGTTTGATTTCACAGTCGTCTCGGGCGTGTGCTCTGACTCTTAAAGCGAAATGACTTGAAAGTACCATGATGTGTTGTTCTTGTAACGGCCTTTCGATCG

**AX131**

CACATCACCCTGTGCCCTCCCAGAACTCCTTTTGGCGTCTGGTGTTTGGGGCGTGGTATATTGGCCCCCCGTGCTCTATGGAGCGCGGTCGGCCTAAAATGACGCACATGACATCTGTCGACACAGAACAGTGGTTGGCGATTAGTCGTTACTATCCTGTGGTGTCGCTTGCGGTGTCGGTTGTTTTGTAGACCCAGAGTGCCGTTGCAACGGTGCCTTGGTTG

**AX132**

CACATCACCCTGTGCCCTCCCAGAACTCCTTTTGGCGTCTGGTGTTTGGGGCGTGGTATATTGGCCCCCCGTGCTCTATGGAGCGCGGTCGGCCTAAAATGACGCACATGACATCTGTCGACACAGAACAGTGGTTGGCGATTAGTCGTTACTATCCTGTGGTGTCGCTTGCGGTGTCGGTTGTTTTGTAGACCCAGAGTGCCGTTGCAACGGTGCCTTGGTTG

**AX141**

CACATCACCCTGTGCCCTCCCAGAACTCCTTTTGGCGTCTGGTGTTTGGGGCGTGGTATATTGGCCCCCCGTGCTCTATGGAGCGCGGTCGGCCTAAAATGACGCACATGACATCTGTCGACACAGAACAGTGGTTGGCGATTAGTCGTTACTATCCTGTGGTGTCGCTTGCGGTGTCGGTTGTTTTGTAGACCCAGAGTGCCGTTGCAACGGTGCCTTGGTTG

**AX151**

CGCATTGTAGCCCCCCACCCTCGTCGTAATGTCTGTGAGGGCTGTGTGGGGCTGATACTGGCCTTCCCGTATGCACAGCAATGCGGTTGGCCCAAATGGAGGAACCCAGGGCGGTGTATGCCATGATGAACGGTGGTGTGTGCTTAGCCTGCCGTCGTTAGAGCATCATGCGCATCATGCCCTTGAGGTATGTTTCGTGGACAACCCCGGTGCAATCATAGCGCGCATCG

**AX161**

CACATCGCCCTGCGCCCTCCCAGCACTCCTTTTGGCGTCTGGTGTCTGGGGCGTGGTATGTTGGCCCCCCGTGCCCCGTGGAGCGCGGTCGGCCTAAAATGGCACACACGACATCTGTCGACACAAAACAGTGGTTGGCGATTAGTCGTTACTATCCTGTGGTGTCGCTtGCGGTGTCGGTTGTCTTGCAGACCCAGAGTGCCGTTGCAACGGTGCCTCGGTTG

**AX171**

CACATCGTTTCCCCAACGCAAACATGTAACAATGTTGCTGCGCGGGGTGTATGCTGACCTCCCGCGAGCACCCGCCTCGTGGTTGGTTGAAATCTGGGTTCATGGCCGACTTCTCCGTGATAAAATGGTGGATGAGCCACGCTCGAGACCAATCACGTGCGAGCCGGTCAGTTGTGGACCCATCGACGACCCTTTGCGTGCACGCACGCTCCCAACG

**AX172**

CGCATCATTGCCCCACCCCACCCCTATGGGGCCAGGTGGTGTGGGCGGAGAATGGCCTCCCGTGAACTACCACTCGCGGTTGGCCCAAATATGAGTACTCGGTGACCAAAGCTGCGACGATCGGTGGTGCAAACATGCCTCTCGAGTTCACGTCGTGTGCCTATGTCTTCCATCAGAGAGACTCAAGGGCCCTTACGCTCTGCAAAAGCAGAGCTCGCATCG

**AX173**

CACATCACCCTGTGCCCTCCCAGAACTCCTTTTGGCGTCTGGTGTTTGGGGCGTGGTATATTGGCCCCCCGTGCTCTATGGAGCGCGGTCGGCCTAAAATGACGCACATGACATCTGTCGACACAGAACAGTGGTTGGCGATTAGTCGTTACTATCCTGTGGTGTCGCTTGCGGTGTCGGTTGTTTTGTAGACCCAGAGTGCCGTTGCAACGGTGCCTTGGTTG

**AX174**

CACAACGTTGCCCCCCCCAACCCTCGCGGTCGAGGGGGCGGAAGATGGCCTCCCGTGAGCTTCTCGCCTCGCGGTTGGCCCAAAAACGAGTCCTCGGCTGCGATCGCCGCGGCATTCGGTGGTTGTCGAAAAATCGGTGCCCCGTCGCGCGCGCTTCTGCAGTTCACGAGCTCCTCAGTACGACCCCAACGCATCGCTCAACGCGGTGCTTCCGACG

**AX181**

CACATCGCCCTGCGCCCTCCCAGCACTCCTTTTGGCGTCTGGTGTCTGGGGCGTGGTATGTTGGCCCCCCGTGCCCCGTGGAGCGCGGTCGGCCTAAAATGGCACACACGACATCTGTCGACACAAAACAGTGGTTGGCGATTAGTCGTTACTATCCTGTGGTGTCGCTTGCGGTGTCGGTTGTCTTGCAGACCCAGAGTGCCGTTGCAACGGTGCCTCGGTTG

**AX191**

CACATCGCCCTGCGCCCTCCCAGCACTCCTTTTGGCGTCTGGTGTCTGGGGCGTGGTATGTTGGCCCCCCGTGCCCCGTGGAGCGCGGTCGGCCTAAAATGGCACACACGACATCTGTCGACACAAAACAGTGGTTGGCGATTAGTCGTTACTATCCTGTGGTGTCGCTTGCGGTGTCGGTTGTCTTGCAGACCCAGAGTGCCGTTGCAACGGTGCCTCGGTTG

**AX201**

CGCATCATTGCCCCACCCCACCCCTATGGGGCCAGGTGGTGTGGGCGGAGAATGGCCTCCCGTGAACTACCACTCGCGGTTGGCCCAAATATGAGTACTCGGTGACCAAAGCTGCGACGATCGGTGGTGCAAACATGCCTCTCGAGTTCACGTCGTGTGCCTATGTCTTCCATCAGAGAGACTCAAGGGCCCTTACGCTCTGCAAAAGCAGAGCTCGCATCG

**AX211**

CACATCGCCCTGCGCCCTCCCAGCACTCCTTTTGGCGTCTGGTGTCTGGGGCGTGGTATGTTGGCCCCCCGTGCCCCGTGGAGCGCGGTCGGCCTAAAATGGCACACACGACATCTGTCGACACAAAACAGTGGTTGGCGATTAGTCGTTACTATCCTGTGGTGTCGCTTGCGGTGTCGGTTGTCTTGCAGACCCAGAGTGCCGTTGCAACGGTGCCTCGGTTG

**AX221**

CACATCGCCCTGCGCCCTCCCAGCACTCCTTTTGGCGTCTGGTGTCTGGGGCGTGGTATGTTGGCCCCCCGTGCCCCGTGGAGCGCGGTCGGCCTAAAATGGCACACACGACATCTGTCGACACAAAACAGTGGTTGGCGATTAGTCGTTACTATCCTGTGGTGTCGCTTGCGGTGTCGGTTGTCTTGCAGACCCAGAGTGCCGTTGCAACGGTGCCTCGGTTG

**AX231**

CGCTTTCGACGCTTCGTCGTTCCCCCCTCGGGGGGTGTGGGGGAACGTGGAGGATGGCCCCCCGTGCCGGAAAGGTGCTGTTGGCCGAAGAGCGGGCCGTCGGTGGTTGTCGAACACGACGCGTGGTGGATGCCTTGTGCGAGCCGTACGTCGTGCCTTCGGGACCCGGGCGAGGCCTCGAGGACCCAAGTAGTGGTGCGAGTCGATGCCTCGGACCG

**AX241**

CACATCGCCCTGCGCCCTCCCAGCACTCCTTTTGGCGTCTGGTGTCTGGGGCGTGGTATATTGGCCCCCCGTGCCCCGTGGAGCGCGGTCGGCCTAAAATGGCACACACGACATCTGTCGACACAAAACAGTGGTTGGCGATTAGTCGTTACTATCCTGTGGTGTCGCTTGCGGTGTCGGTTGTCTTGCAGACCCAGAGTGCCGTTGCAACGGTGCCTCGGTTG

**AX251**

CGCATCGTCGCCCCCTCAACCCCACGCCTCGAGCGAGGCGCGGGGACACGTTAGGGGCGGATATTGGCCTCCCGTGGGACGCTTAATCCCGCGGTTGGCCCAAATACGAGTCCTCGGCGTCGGACGCCGCGACGTTCGGTGGTGGAAAAAGTAAGAAACCTCGAGCTCGCGTCGCGCGTACGTCGTCGGTTTAAGGCTCCCGACCCTGAACGCATCG

**AX261**

CACATCACCCTGTGCCCTCCCAGAACTCCTTTTGGCGTCTGGTGTTTGGGGCGTGGTATATTGGCCCCCCGTGCTCTATGGAGCGCGGTCGGCCTAAAATGACGCACATGACATCTGTCGACACAGAACAGTGGTTGGCGATTAGTCGTTACTATCCTGTGGTGTCGCTTGCGGTGTCGGTTGTTTTGTAGACCCAGAGTGCCGTTGCAACGGTGCCTTGGTTG

**AX271**

CACATCGCCCTGCGCCCTCCCAGCACTCCTTTTGGCGTCTGGTGTCTGGGGCGTGGTATGTTGGCCCCCCGTGCCCCGTGGAGCGCGGTCGGCCTAAAATGGCACACACGACATCTGTCGACACAAAACAGTGGTTGGCGATTAGTCGTTACTATCCTGTGGTGTCGCTTGCGGTGTCGGTTGTCTTGCAGACCCAGAGTGCCGTTGCAACGGTGCCTCGGTTG

**Raw secondary structure**

**AX011**

......(((...((((((.(((((...((....))..))))).....)))))).))).....((((..((((((((....))))))))..))))....((((.(((..(((((.((((((((((...((((.(((((((((....))))))..))))))).))))))...)))))))))..))).)))).((((.((.(((((((....))))))))).)))).

**AX012**

.......(((...((((((.(((((...((....))..))))).....)))))).))).....((((..((((((((....))))))))..))))....((((.(((..(((((((.(.((((((...((((.(((((((((....))))))..))))))).)))))))....)))))))..))).))))....((((.(((((((....))))))).))))

**AX013**

......(((...((((((.(((((...((....))..))))).....)))))).))).....((((..((((((((....))))))))..))))....((((.(((..(((((.((((((((((...((((.(((((((((....))))))..))))))).))))))...)))))))))..))).)))).((((.((.(((((((....))))))))).)))).

**AX014**

......(((...((((((.(((((...((....))..))))).....)))))).))).....((((..((((((((....))))))))..))))....((((.(((..(((((.((((((((((...((((.(((((((((....))))))..))))))).))))))...)))))))))..))).)))).((((.((.(((((((....))))))))).)))).

**AX021**

.........(((((((((.((((.((.((....)).)))))).....)))))))))......((((..((((((((...)).))))))..)))).....(((.(((..(((((.((((((((((...((((.(((((((((....))))))..))))))).))))))...)))))))))..))).)))..((((.((.(((((((....))))))))).)))).**AX031**

.........(((((((((.((((.((.((....)).)))))).....)))))))))......((((..((((((((...)).))))))..)))).....(((.(((..(((((.((((((((((...((((.(((((((((....))))))..))))))).))))))...)))))))))..))).)))..((((.((.(((((((....))))))))).)))).

**AX041**

.........(((((((((.((((.((.((....)).)))))).....)))))))))......((((..((((((((...)).))))))..)))).....(((.(((..(((((.((((((((((...((((.(((((((((....))))))..))))))).))))))...)))))))))..))).)))..((((.((.(((((((....))))))))).)))).**AX051**

.........(((((((((.((((.((.((....)).)))))).....)))))))))......((((..((((((((...)).))))))..)))).....(((((((..(((((.((((((((((...((((.(((((((((....))))))..))))))).))))))...)))))))))..)))))))..((((.((.(((((((....))))))))).)))).**AX061**

.........(((((((((.((((.((.((....)).)))))).....)))))))))......((((..((((((((...)).))))))..)))).....(((.(((..(((((.((((((((((...((((.(((((((((....))))))..))))))).))))))...)))))))))..))).)))..((((.((.(((((((....))))))))).)))).

**AX062**

.........(((((((((.((((.((.((....)).)))))).....)))))))))......((((..((((((((...)).))))))..)))).....(((.(((..(((((.((((((((((...((((.(((((((((....))))))..))))))).))))))...)))))))))..))).)))..((((.((.(((((((....))))))))).)))).

**AX071**

..(.((..(((((((..(((((.(.((...)).).).)......).))))))))))).).((.((..((((((((...)).))))))..))))..........(((..(.((.((((((((((((((((((((((.(.(((.((...)).))))))))))....))))))).)))).))))).))....)..)))(((((....(.....((((...(...).)))).))))))

**AX072**

......(((...((((((.(((((...((....))..))))).....)))))).))).....((((..((((((((....))))))))..))))....((((.(((..(((((.((((((((((...((((.(((((((((....))))))..))))))).))))))...)))))))))..))).)))).((((.((.(((((((....))))))))).)))).

**AX081**

......(((...((((((.(((((...((....))..))))).....)))))).))).....((((..((((((((....))))))))..))))....((((.(((..(((((.((((((((((...((((.(((((((((....))))))..))))))).))))))...)))))))))..))).)))).((((.((.(((((((....))))))))).)))).

**AX091**

......(((...((((((.(((((...((....))..))))).....)))))).))).....((((..((((((((....))))))))..))))....((((.(((..(((((.((((((((((...((((.(((((((((....))))))..))))))).))))))...)))))))))..))).)))).((((.((.(((((((....))))))))).)))).

**AX092**

......(((...((((((.(((((...((....))..))))).....)))))).))).....((((..((((((((....))))))))..))))....((((.(((..(((((.((((((((((...((((.(((((((((....))))))..))))))).))))))...)))))))))..))).)))).((((.((.(((((((....))))))))).)))).

**AX093**

......(((...((((((.(((((...((....))..))))).....)))))).))).....((((..((((((((....))))))))..))))....((((.(((..(((((.((((((((((...((((.(((((((((....))))))..))))))).))))))...)))))))))..))).)))).((((.((.(((((((....))))))))).)))).**AX101**

......(((...((((((.(((((...((....))..))))).....)))))).))).....((((..((((((((....))))))))..))))....((((.(((..(((((.((((((((((...((((.(((((((((....))))))..))))))).))))))...)))))))))..))).)))).((((.((.(((((((....))))))))).)))).**AX102**

........(((((((.(.((((((.((((((....)))))).)))).....)))))))))).....((((..((((((((.......))))))))..)))).......(((((.((((((.((.((((.(((((((((((.(((........(...).)))))))....))))))))))).)).)))))).....)))))...........(((...))).

**AX111**

.........(((((((((.((((.((.((....)).)))))).....)))))))))......((((..((((((((...)).))))))..)))).....(((.(((..(((((.((((((((((...((((.(((((((((....))))))..))))))).))))))...)))))))))..))).)))..((((.((.(((((((....))))))))).)))).

**AX121**

.........(((((((((.((((.((.((....)).)))))).....)))))))))......((((..((((((((...)).))))))..)))).....(((.(((..(((((.((((((((((...((((.(((((((((....))))))..))))))).))))))...)))))))))..))).)))..((((.((.(((((((....))))))))).)))).

**AX122**

..........(((((((((((((((((((......))).))))).).)))))))))).....((((..(((((((...)))))))..)))).......(((.(((.(((((((...((((((..(.((((((.(((((..(((...((((.((((((....)).))))........))))..))).)))))..))))))).)))))).))))).)).))).)))(((....)))

**AX131**

......(((...((((((.(((((...((....))..))))).....)))))).))).....((((..((((((((....))))))))..))))....((((.(((..(((((.((((((((((...((((.(((((((((....))))))..))))))).))))))...)))))))))..))).)))).((((.((.(((((((....))))))))).)))).**AX132**

......(((...((((((.(((((...((....))..))))).....)))))).))).....((((..((((((((....))))))))..))))....((((.(((..(((((.((((((((((...((((.(((((((((....))))))..))))))).))))))...)))))))))..))).)))).((((.((.(((((((....))))))))).)))).

**AX141**

......(((...((((((.(((((...((....))..))))).....)))))).))).....((((..((((((((....))))))))..))))....((((.(((..(((((.((((((((((...((((.(((((((((....))))))..))))))).))))))...)))))))))..))).)))).((((.((.(((((((....))))))))).)))).**AX151**

..(.((..(((((((..(((((.(.((...)).).).)......).))))))))))).).((.((..((((((((...)).))))))..))))..........(((..(.((.((((((((((((((((((((((.(.(((.((...)).))))))))))....))))))).)))).))))).))....)..)))(((((....(.....((((...(...).)))).))))))

**AX161**

.........(((((((((.((((.((.((....)).)))))).....)))))))))......((((..((((((((...)).))))))..)))).....(((.(((..(((((.((((((((((...((((.(((((((((....))))))..))))))).))))))...)))))))))..))).)))..((((.((.(((((((....))))))))).)))).**AX171**

......(((..((((..(((((((((......)))))..)))).))))...)))..((((..((((((((....).)))))))..))))......((((((((((((((..(((((((((((....((((...(((((...)))))..)))))))))).))))).))))))....)))))))).............(((((....))))).......

and

......(((..((((..(((((((((......)))))..)))).))))...)))..((((..((((((((....).)))))))..))))......((((((((((((((..(((((((((((....((((...(((((...)))))..)))))))))).))))).))))))....)))))))).............(((((....))))).........

**AX172**

..((.((.((((((((((((.((((...))))...)).)))).)))))).))...))...((((((........))))))..(((((.....(((((.(((((.(((....(((((((((.((((.((.(((....))))).)))).....))))))).))....)))..))....)))..))))).))))).....(((((((....))))))).........

**AX173**

......(((...((((((.(((((...((....))..))))).....)))))).))).....((((..((((((((....))))))))..))))....((((.(((..(((((.((((((((((...((((.(((((((((....))))))..))))))).))))))...)))))))))..))).)))).((((.((.(((((((....))))))))).)))).**AX174**

........((((((((.(((((.....))))).)))))))).....((((..(((((((((...)).)))))))..))))......((((..(((((((((((.(((((((((....((.(((..((((....)))).))))))))))).))).))..)))))...))))...))))..............((((((((...))))))))...........

**AX181**

.........(((((((((.((((.((.((....)).)))))).....)))))))))......((((..((((((((...)).))))))..)))).....(((.(((..(((((.((((((((((...((((.(((((((((....))))))..))))))).))))))...)))))))))..))).)))..((((.((.(((((((....))))))))).)))).**AX191**

.........(((((((((.((((.((.((....)).)))))).....)))))))))......((((..((((((((...)).))))))..)))).....(((.(((..(((((.((((((((((...((((.(((((((((....))))))..))))))).))))))...)))))))))..))).)))..((((.((.(((((((....))))))))).)))).**AX201**

..((.((.((((((((((((.((((...))))...)).)))).)))))).))...))...((((((........))))))..(((((.....(((((.(((((.(((....(((((((((.((((.((.(((....))))).)))).....))))))).))....)))..))....)))..))))).))))).....(((((((....))))))).........

**AX211**

.........(((((((((.((((.((.((....)).)))))).....)))))))))......((((..((((((((...)).))))))..)))).....(((.(((..(((((.((((((((((...((((.(((((((((....))))))..))))))).))))))...)))))))))..))).)))..((((.((.(((((((....))))))))).)))).**AX221**

.........(((((((((.((((.((.((....)).)))))).....)))))))))......((((..((((((((...)).))))))..)))).....(((.(((..(((((.((((((((((...((((.(((((((((....))))))..))))))).))))))...)))))))))..))).)))..((((.((.(((((((....))))))))).)))).**AX231**

...((((((.((.((((((.((((((((...))))))).))))))))))))))).(((((..(((((((.....)))))))..)))))......(((((.(((.(((((.((((((((((((.(((((...(((.....))).))))).)))))))..))))))))).)..))))))))............((((((((((...)).))))))))...

**AX241**

.........(((((((((.((((.((.((....)).)))))).....)))))))))......((((..((((((((...)).))))))..)))).....(((.(((..(((((.((((((((((...((((.(((((((((....))))))..))))))).))))))...)))))))))..))).)))..((((.((.(((((((....))))))))).)))).

**AX251**

........(((((((.(.((((((.((((((....)))))).)))).....)))))))))).....((((..((((((((.......))))))))..)))).......(((((.((((((.((.((((.(((((((((((.(((........(...).)))))))....))))))))))).)).)))))).....)))))...........(((...))).

**AX261**

......(((...((((((.(((((...((....))..))))).....)))))).))).....((((..((((((((....))))))))..))))....((((.(((..(((((.((((((((((...((((.(((((((((....))))))..))))))).))))))...)))))))))..))).)))).((((.((.(((((((....))))))))).)))).**AX271**

.........(((((((((.((((.((.((....)).)))))).....)))))))))......((((..((((((((...)).))))))..)))).....(((.(((..(((((.((((((((((...((((.(((((((((....))))))..))))))).))))))...)))))))))..))).)))..((((.((.(((((((....))))))))).)))).
